# Regulation of virulence in *Chromobacterium violaceum* and strategies to combat it

**DOI:** 10.3389/fmicb.2024.1303595

**Published:** 2024-01-24

**Authors:** Mahendrarajan Venkatramanan, Easwaran Nalini

**Affiliations:** Department of Integrative Biology, School of Biosciences and Technology, Vellore Institute of Technology, Vellore, India

**Keywords:** *Chromobacterium violaceum*, quorum sensing, immune effects, bio actives, quorum quenching

## Abstract

*Chromobacterium* is a rod-shaped, Gram-negative, facultatively anaerobic bacteria with a cosmopolitan distribution. Just about 160 *Chromobacterium violaceum* incidents have been reported globally, but then once infected, it has the ability to cause deadly septicemia, and infections in the lungs, liver, brain, spleen, and lymphatic systems that might lead to death. *C. violaceum* produces and utilizes violacein to kill bacteria that compete with it in an ecological niche. Violacein is a hydrophobic bisindole that is delivered through an efficient transport route termed outer membrane vesicles (OMVs) through the aqueous environment. OMVs are small, spherical segments detached from the outer membrane of Gram-negative bacteria. *C. violaceum* OMV secretions are controlled by a mechanism called the quorum sensing system *CviI*/*CviR*, which enables cell-to-cell communication between them and regulation of various virulence factors such as biofilm formation, and violacein biosynthesis. Another virulence factor bacterial type 3 secretion system (T3SS) is divided into two types: *Cpi-1* and *Cpi-2*. *Cpi-1*’s needle and rod effector proteins are perhaps recognized by NAIP receptors in humans and mice, activating the NLRC4 inflammasome cascade, effectively clearing spleen infections via pyroptosis, and cytotoxicity mediated by IL-18-driven Natural killer (NK) cells in the liver. In this paper, we attempt to interrelate quorum-controlled biofilm formation, violacein production, violacein delivery by OMVs and T3SS effector protein production and host mediated immunological effects against the *Cpi1* of T3SS. We suggest a research path with natural bioactive molecule like palmitic acid that can act as an anti-quorum agent by reducing the expression of virulence factors as well as an immunomodulatory agent that can augment innate immune defense by hyperactivation of NLRC4 inflammasome hence dramatically purge *C. violaceum* infections.

## Introduction

*Chromobacterium violaceum* is a beta proteobacterium that forms violet-colored colonies with smooth surface. *C. violaceum* is a facultative anaerobe and a non-sporing bacillus (Kumar, 2012). They are motile with a single flagellum at a pole, one to four lateral flagella as well as pili all over the bacterium liable for the motility (Ravichandran et al., 2018). Trehalose, gluconate, glucose, and N-acetylglucosamine are fermentable by *C. violaceum*.

*C. violaceum* is positive for oxidase, which converts oxygen to water in the presence of cytochrome c and proton, and also catalase, which converts hydrogen peroxide to water and oxygen (Antony et al., 2013). Saprophytic *C. violaceum* can be figured in soil and water across the planet with predominant presence in the tropical and subtropical regions (Batista and da Silva Neto, 2017). Malaysia, Brazil, Japan, Sri Lanka, Taiwan, United States, Singapore, Argentina, Nigeria, Vietnam, Australia, Canada, Cuba, and India reported numerous *C. violaceum* infection cases. They exist as natural microbiota of water and soil, but the most exemplary way for them to enter the bloodstream and cause systemic infections is through crippled wounds or cuts in the skin, where the bacterium invades from a polluted surface or water (Alisjahbana et al., 2021). Various case studies involving *C. violaceum* cognate complications such as bacterial hemophagocytic syndrome, brain abscess, chronic cellulitis, conjunctivitis, chronic granulomatosis, diarrhoea, endocarditis, internal jugular vein thrombophlebitis, meningitis, neutropenic sepsis, orbital cellulitis, osteomyelitis, pneumonia, puerperal sepsis, retropharyngeal infection, septic spondylitis and urinary tract infections, are mostly reported in immunocompromised individuals (Lin et al., 2016). The genetic material of bacteria is a single circular chromosome of 4751.8 kb in which the percentage of GC is roughly 64.83. *C. violaceum*’*s* genome has a broad but incomplete array of open reading frames (ORFs) that code for mammalian pathogenicity-associated proteins. This could be the cause of the high lethality rate but with infrequent pathogenicity in humans. The size of *C. violaceum* bacterial cells may vary from 0.6 to 0.9 μm & 1.5 to 3.0 μm. Temperatures of 30–37 degrees Celsius in both aerobic and anaerobic environments are optimum for promoting growth *in vitro*, yet, the anaerobic state causes the depletion in pigment violacein synthesis. The appropriate pH is 4, however, pH below or equal to 3 is decidedly inimical to the bacteria (Castro et al., 2015). Apart from the familiar antibiotic pigment violacein, they are capable of producing extended antibiotics such as aerocyanidin, aerocavin which are highly decisive against both Gram positive and negative organisms and aztreonam which is decisive against Gram negative bacteria (Parker et al., 1988). In addition to humans, it can infect other mammal species such as pigs, sheep, dogs, buffaloes, and monkeys (Liu et al., 2012). Ciprofloxacin is the most effective antibiotic, followed by norfloxacin and perfoxacin against *C. violaceum* (Nayyar et al., 2019).

The first episode of *C. violaceum* in India was observed in an 11 months-old boy who died of septicemia within 48 h. The second instance is a 2 years-old male infant who died barely 48 h well before the antibiotic susceptibility test was completed. Both of these fatal cases describe the severity of the *C. violaceum* infection. It can also cause a moderate infection which is not detrimental to life, such as in the third case, which involves a 12 years-old female with urinary tract infection. The infected region is characterized by ulcerated lesions that exude a bluish purulent fluid and are mobbed by inflammation. More than 10% of *C. violaceum* infections occur in patients suffering from chronic granulomatous illness. Hepatitis, or liver inflammation, is perhaps the most common human manifestation of *C. violaceum* infection (Alisjahbana et al., 2021).

Since multidrug resistance (MDR) is ubiquitous amongst harmful microbes, treating infectious microbes like *C. violaceum* has become a tedious task. Anti-quorum sensing and immunomodulation can be combined to combat bacterial infections, such as those caused by *C. violaceum*. The aim of this review is to shed light on the dual mode therapy, where a single drug molecule should be utilized for halting virulence of infectious microbes as well as host immunomodulation that can boost the immune cells to clear the infection efficiently. Thus, the development of antibiotic resistance can be lessened to some extent. *C. violaceum* is a model organism for anti-quorum experiments as they produce violacein pigment. So, we focussed on *C. violaceum* alone and proposed a possible mechanism to fight against them which can be further extended to other pathogenic microorganisms.

### Clinical significance

**Figure 1:**
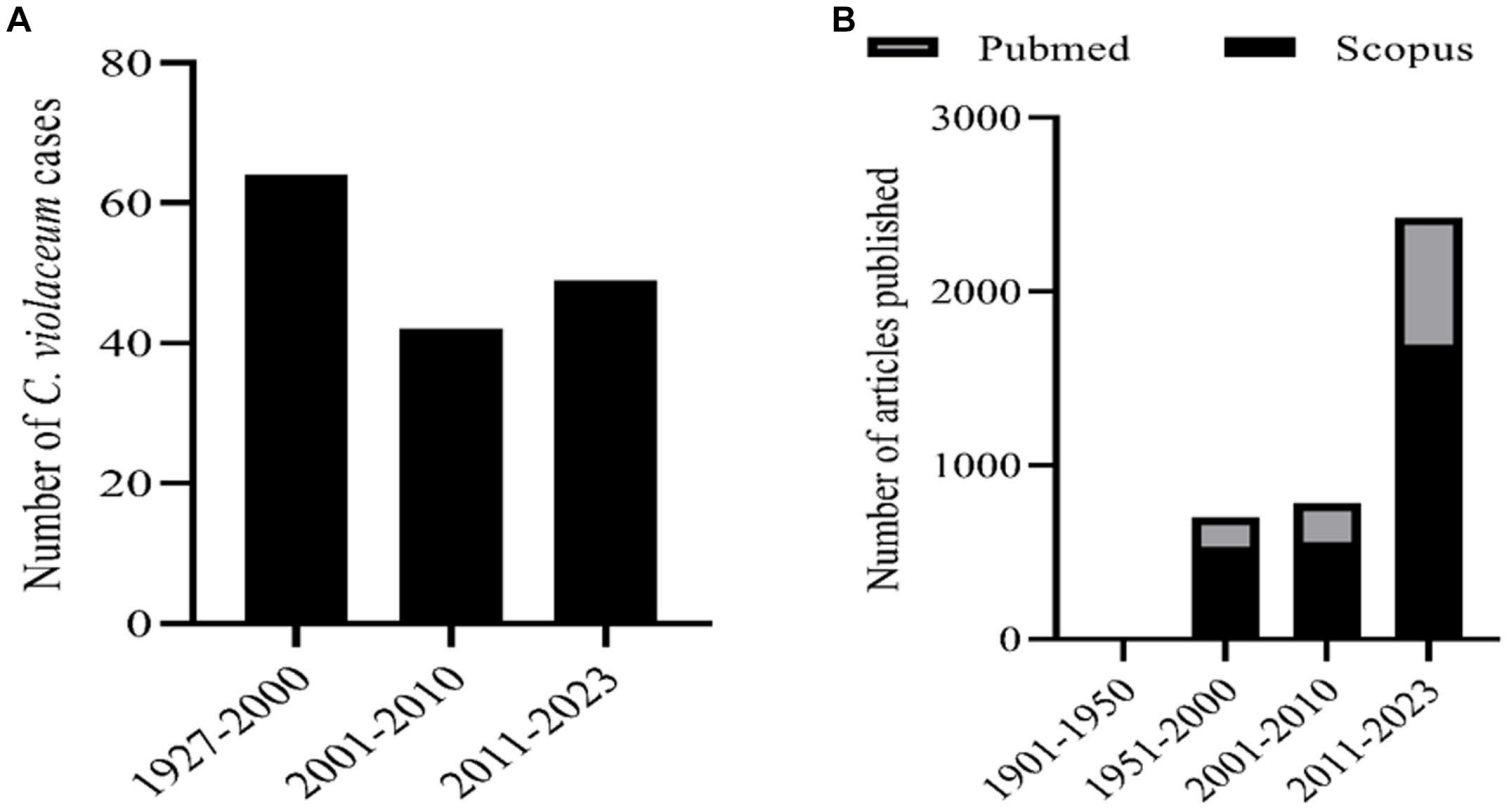
**(A)** Number of human infection cases of *C. violaceum* infections. **(B)** Number of articles related with *C. violaceum* in Scopus and PubMed.

*C. violaceum* infections are clinically significant as they can lead to fatal outcomes if not diagnosed and treated promptly. The diagnosis of *C. violaceum* infection is based on the isolation and identification of the bacterium from clinical specimens, such as blood, pus, urine, or cerebrospinal fluid. The treatment of *C. violaceum* infection requires a combination of antibiotics, surgical drainage, and supportive care. The mortality rate of *C. violaceum* infection is high, ranging from 20 to 60%, depending on the severity and location of the infection. As discussed earlier there were more than 160 cases reported globally in humans. But if we notice the infection pattern from the year of 1927 to 2000 in the span of nearly 70 years the infection cases were only 64. But in the decade of 2001 to 2010 the infected cases were 42 which further escalated in the last decade to 49 cases (Figure 1A). The antibiotic resistant pattern of *C. violaceum* depicts that the infection rate might further increase in the following years over irregular treatment strategies. The antibiotic sensitivity/resistance pattern is depicted in Table 1. Among the 37 antibiotics screened in our laboratory and from previous literature, *C. violaceum* was found to be resistant to 15 antibiotics, intermediate resistance to 6 antibiotics which explains the difficulty in treatment strategies. Moreover the *C. violaceum* bacteria is of high research interest because of the ability to serve as a quorum sensing model organism for various other severe pathogenic bacterial species because of its violacein pigment production. If we look into the publication count in the databases such as Scopus and PubMed on *C. violaceum*, the number drastically increases over the last as well as the previous decade, (Figure 1B). Thus targeting this bacterium to find novel therapy can be beneficial in many ways to treat *C. violaceum* and other clinically significant pathogenic species.

**Table 1:** Antibiotic resistance/sensitivity pattern of *C. violaceum*.

S. No.	Name of the antibiotic	Concentration in μg/mL	Resistance/sensitivity pattern
1.	Amikacin	30	−
2.	Amoxycillin	10	±
3.	Ampicillin	25	+
4.	Azithromycin	15	−
5.	Aztreonam	50	+
6.	Carbamycin	50	+
7.	Cefadrozil	30	+
8.	Cefoperazone	75	±
9.	Ceftazidime	30	±
10.	Ceftriaxone	30	−
11.	Cefuroxime	30	+
12.	Cephotaxime	30	+
13.	Chloramphenicol	10	−
14.	Ciprofloxacin	5	−
15.	Cloxacillin	1	+
16.	Co-trimoxazole	25	−
17.	Erythromycin	50	+
18.	Ethidium bromide	60	+
19.	Fusidic acid	10	+
20.	Gentamicin	10	−
21.	Imipenem	10	−
22.	Kanamycin	30	−
23.	Meropenem	10	+

(+), Resistant; (−), Sensitive; (±), Intermediate. Data was presented with reference (Dal’Molin et al., 2009; Kothari et al., 2017) and data obtained from our lab.

### Quorum sensing regulations

Quorum sensing allows bacteria to communicate and coordinate its behavior according to their population density. It includes synthesis and detection of chemical signals called autoinducers (AI). Autoinducers can diffuse through the cell membrane and bind the corresponding receptors (Rutherford and Bassler, 2012). Gram negative bacteria use acyl homoserine lactone (AHL) as autoinducers which are derived from S-adenosylmethionine (SAM). AHLs varies in structure and length in its acyl chain which determine its activity and specificity in binding. AHLs can regulate various functions such as bioluminescence, virulence production, antimicrobial resistance (AMR) and biofilm formation. Quorum sensing involve two types of receptors called cytoplasmic transcription factors and membrane bound histidine sensor kinases. The transcription factors are often the members of LuxR family which bind to AHLs and DNA. The sensor kinases are part of two component system that transmit signals from AI to transcription factors. Quorum sensing can serve as a potential target for developing new strategies to control infectious pathogens by interfering with quorum sensing receptor or associated signals (Papenfort and Bassler, 2016).

The first identified quorum sensing pathway in Gram-negative bacteria is the LuxI-LuxR system in *Vibrio fischeri*, a marine bacterium that displays luminescence. The LuxI protein is an AHL synthase that converts S-adenosylmethionine (SAM) into N-(3-oxohexanoyl)-L-homoserine lactone (3OC6-HSL), which is the autoinducer for this system. The LuxR protein is a transcriptional regulator that binds to 3OC6-HSL and activates the expression of the lux operon, which encodes the enzymes for bioluminescence. The LuxI-LuxR system is an example of autoinduction, where the detection of the autoinducer stimulates its own production, creating a positive feedback loop (Lupp and Ruby, 2005).

Another example of a quorum sensing pathway in Gram-negative bacteria is the LasI-LasR system in *Pseudomonas aeruginosa*, an opportunistic pathogen that is clinically important. The LasI protein is an AHL synthase that converts SAM into N-(3-oxododecanoyl)-L-homoserine lactone (3OC12-HSL), which is the autoinducer for this system. The LasR protein is a transcriptional regulator that binds to 3OC12-HSL and activates or represses the expression of various genes, including those involved in virulence and antibiotic resistance. The LasI-LasR system is an example of signal integration, where multiple autoinducers and receptors work together to coordinate the bacterial behavior. For instance, *P. aeruginosa* also produces another AHL called N-butanoyl-L-homoserine lactone (C4-HSL) through the RhlI-RhlR system, which interacts with the LasI-LasR system to fine-tune the expression of quorum sensing-regulated genes (Miranda et al., 2022). The RhlI and RhlR quorum system regulates rhamnolipid synthesis and virulence protein expression in the infected host cytoplasm. The Rhl system regulates the biofilm formation and development. The third QS system called Pseudomonas Quinolone System (PQS) is associated with the biosynthesis of 2-heptyl-3-hydroxy-4-quinolone signal by ABCDE operon. PQS signaling molecule transported via outer membrane vesicles promotes iron sequestration. PQS system also govern the formation of rhamnolipid and associated biofilm. The recently identified IQS quorum sensing system produces 2-(2-hydroxyphenyl)-thiazole-4-carbaldehyde as its signal molecule. However, the molecular mechanism behind its signaling and the genes regulated by the IQS system has not yet discovered. Thus, *P. aeruginosa* interlinked quorum systems such as *Las*, *Rhl*, *PQS* and *IQS* cause the greatest of threats in human infection cases (Vadakkan et al., 2024).

### Quorum sensing in *Chromobacterium violaceum*

**Figure 2:**
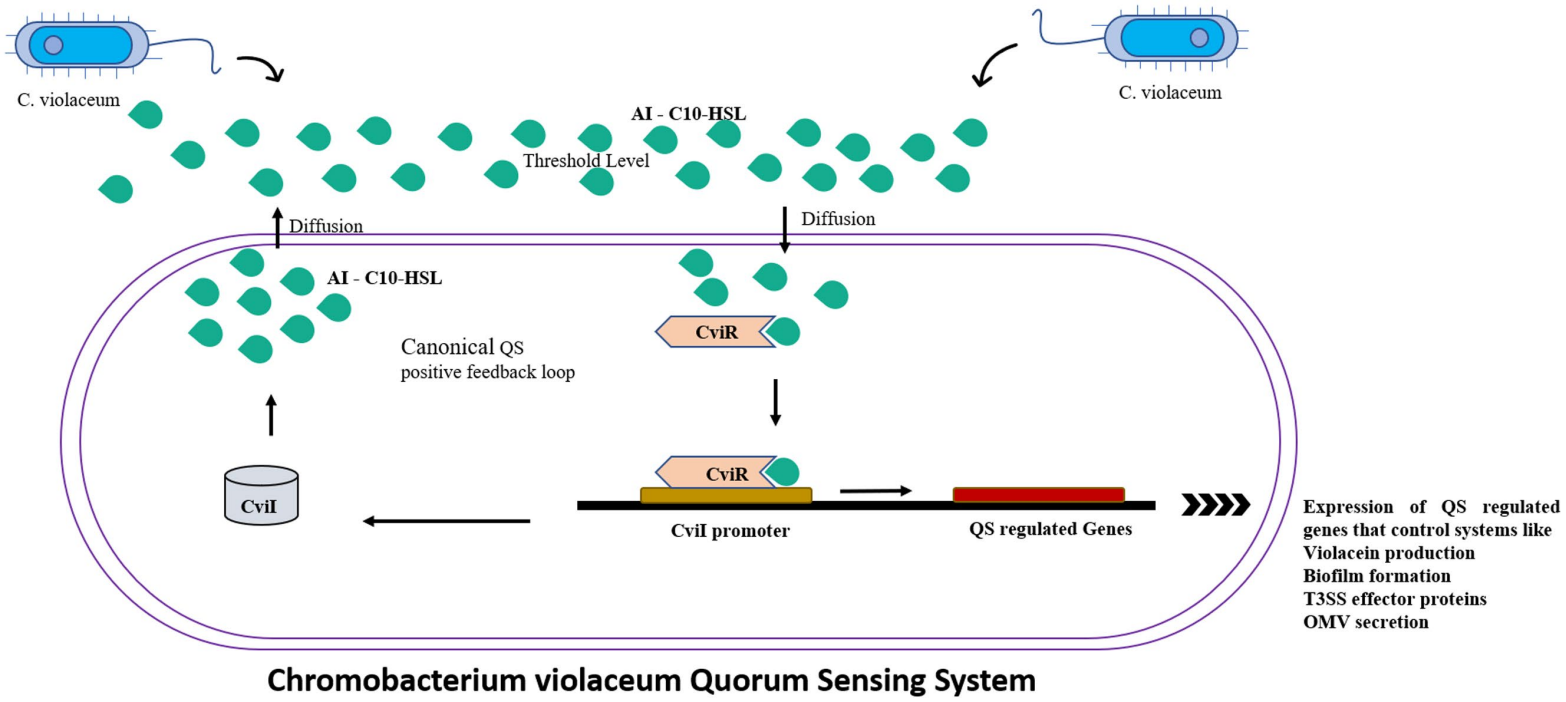
The canonical positive feedback loop is employed by *C. violaceum*. Autoinducer C10-HSls were secreted by *CviI* synthase which when reached the threshold level detected by the neighboring bacteria’s *CviR*, which is a DNA binding transcriptional regulator which controls the quorum sensing regulated systems such as violacein production, biofilms and outer membrane vesicles.

*C. violaceum* incorporates a huge number of virulence mechanisms, one of which is the quorum-sensing system (Ciprandi et al., 2013). Quorum sensing is a type of cell-to-cell communication that aid bacteria to communicate with one another (Castro-Gomes et al., 2014). The basic quorum sensing mechanism in *C. violaceum* is represented in Figure 2. Autoinducers are tiny chemical molecules responsible for the transmission of quorum signals between them (Høiby et al., 2010). Quorum sensing in *C. violaceum* is mediated by two genes, *cviI* and *cviR*. They are analogous to the *luxI* and *luxR* homologues of *V. fischeri*, and responding with high affinity towards AHL (acyl homoserine lactone). *CviI* is an AHL synthase gene that governs the biosynthesis of N-decanoyl L-homoserine lactone (C10-HSL), a signaling molecule. CviR is a transcriptional regulator protein that governs gene expression following binding to CviI product. These two genes are adjacent neighbours; however, transcription tends to take place on different DNA strands that express overlapping regions up to 73 bp in length. When coupled to CviR, the chain length of C4 to C8 AHL molecules stimulates *vioA* transcription responsible for the synthesis of violacein. On the other hand, AHL with chain lengths of C10 to C14 is an inactive antagonist (Høiby et al., 2010). The AHL molecule attaches to the cognate receptor, which as a complex regulates the expression of any of the target genes as the bacterial population increases (Stauff and Bassler, 2011). When there is no threshold population, AHL remains at a low concentration, delaying the onset of signal receptor complexes. At the same time, the unbound unstable CviR dissociates and therefore unable to bind to its palindrome sequence binding site CTGNCCNNNNGGNCAG (Castro-Gomes et al., 2014). When compared to TraR, a receptor protein for 3 oxo C8 HSL in *Agrobacterium tumefaciens*, CviR domains are fairly similar, but the organization is clearly distinct. Unlike TraR, CviR and chlorolactone (CL) an inhibitor of CviR have a cross subunit architecture wherein every monomer’s DNA binding domain is stationed underneath the ligand-binding domain of the opposite monomer, showcasing LBD-DBD interactions. They result in a 60-degree separation between two DNA binding helices. The space required for efficient operator binding is 30 Å, so the declined DNA binding affinity of the CviR: CL complex is fairly understood. CviR full-length structure is complex with its antagonist so this cross-subunit structure may result from that interaction. Thus the proposed hypothesis of CviR inhibition by CL bound to autoinducer cavity and the induction of closed confirmation caused the inability of DNA to bind is acknowledged (Brumbach et al., 2007).

### Biofilm formation and signal transduction mechanism regulated by quorum sensing

#### Biofilms

**Figure 3:**
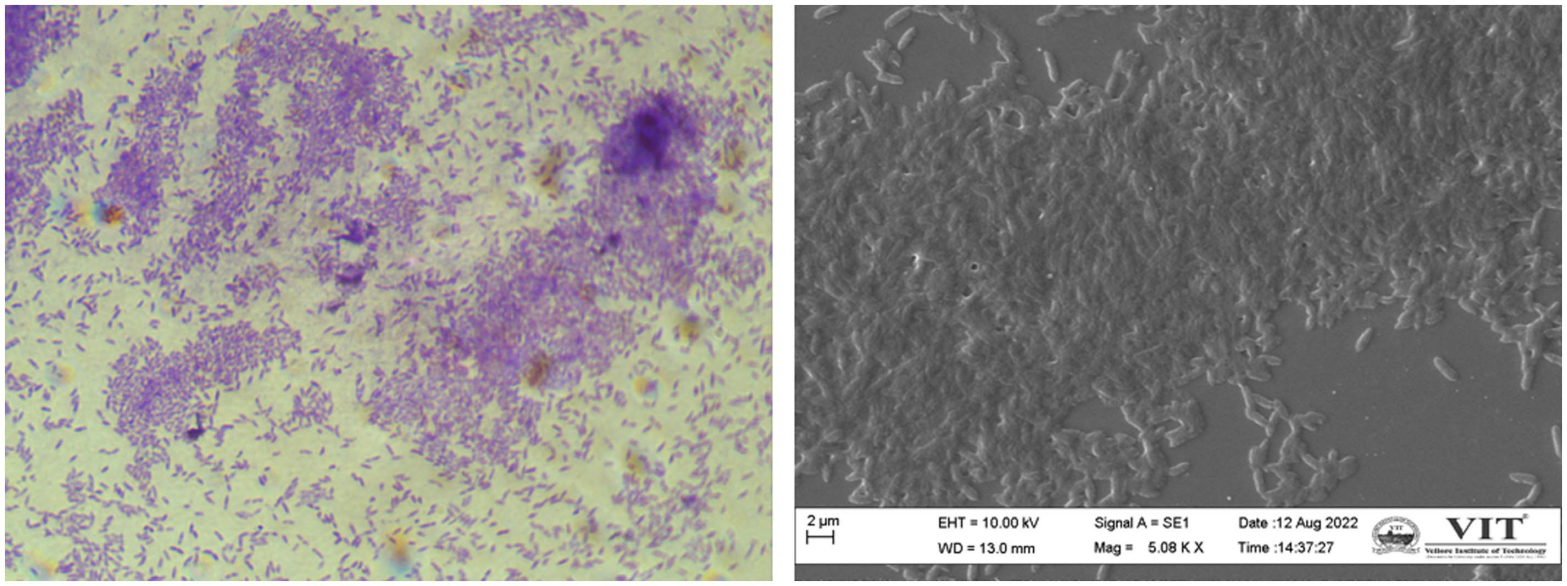
*Chromobacterium violaceum* biofilms formation is regulated by quorum sensing **(A)** compound microscopy **(B)** scanning electron microscopy. The images were obtained in our laboratory using a compound light microscope and a Carl Zeiss Evo/18 scanning electron microscope.

Depending entirely on the bacterial population, biofilms are formed and regulated in the same population-dependent manner by *C. violaceum*’*s hmsHNFR* gene (Becker et al., 2009). Biofilm images of compound light and scanning electron microscope (SEM) portrayed in Figure 3 were data from our lab. The synthesis of virulence factors in multilayer biofilm aids infection and makes bacteria immensely harmful. Biofilms facilitate safeguarding microorganisms from a range of environmental and artificial stress and strain, such as pH, temperature, antibiotics, antimicrobials, and many more. Biofilm greatly help to establish bacterial adhesion and allows survival in harsh habitat. As a result, destroying it, will be a pivotal stage in the biofilm-forming severe bacterial infections of *C. violaceum* (Yin et al., 2019).

#### Violacein

Violacein, a purple pigment, is a hydrophobic moiety with antimicrobial characteristics that gives the typical violet-coloured colonies of *C. violaceum*. Its synthesis is constrained by the quorum-sensing machinery of *C. violaceum*, which is population-dependent. Violacein production is influenced by a multitude of genes, including *vioA, vioB*, *vioC*, *vioD* and *vioE*, all of which are transcribed in almost the same way and encoded by 7.3 kb DNA segments (McClean et al., 1997). The *vioABCDE* operon governs the biosynthesis of violacein from the amino acid tryptophan. The CviI synthase enzyme contributes towards the conversion of fatty acids or S adenosyl methionine to AHLs, which further forms a complex with the CviR and stimulates the *vioABCDE* operon (McClean et al., 1997). In *C. violaceum*, the regulatory domain of CviR regulates *vioA* and other specific promoters (Swem et al., 2009; Høiby et al., 2010).

**Figure 4:**
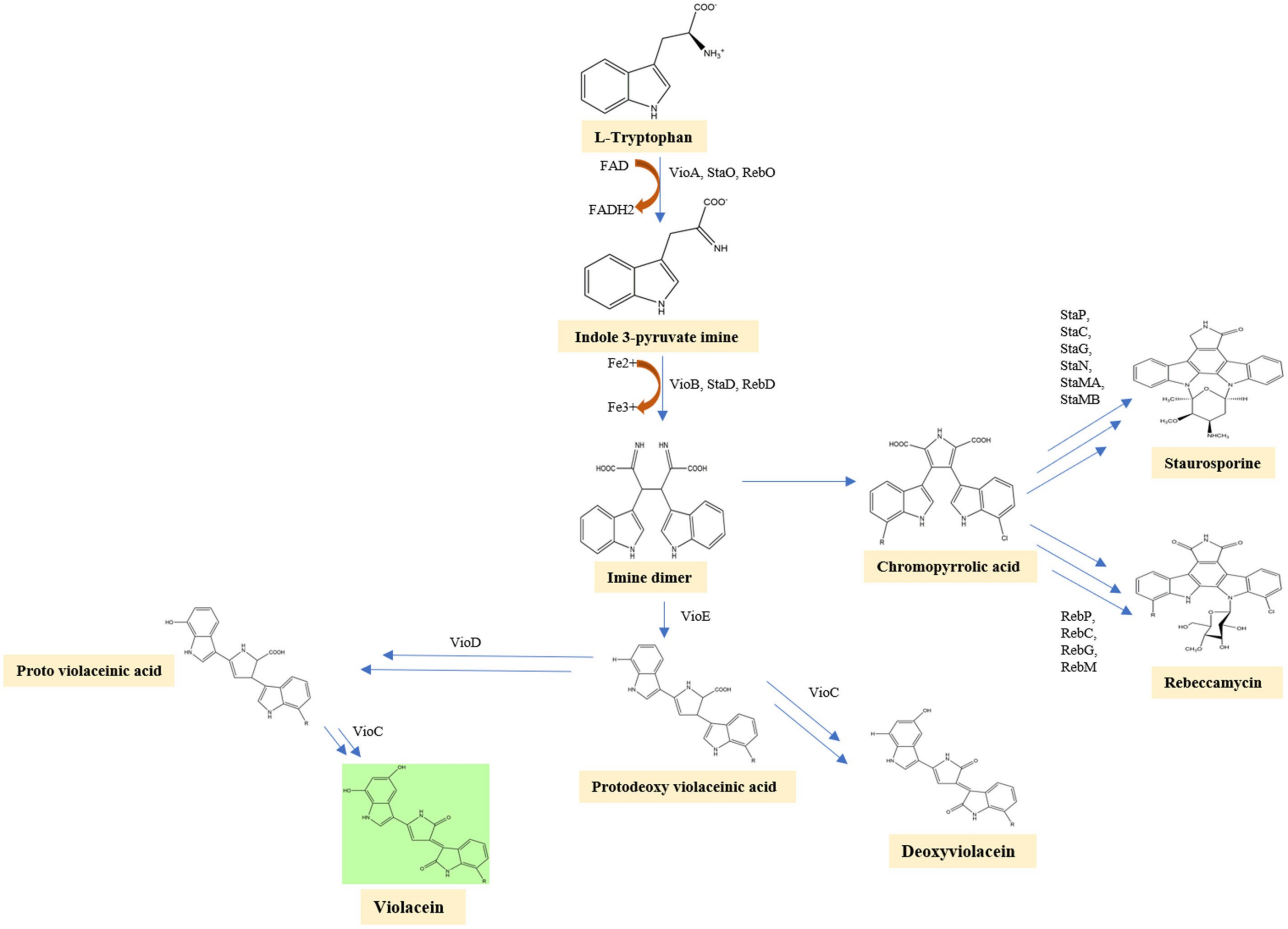
In the first step of the violacein synthesis, the enzyme VioA oxidizes L-tryptophan to generate indole-3 pyruvic acid imine. The IPA imine is then dimerized utilizing the enzymes heme-containing oxidase VioB, StaD, and RebD to form an unstable molecule imine dimer. VioE converts this unstable imine dimer into protodeoxyviolaceinic acid. VioD, a flavin-dependent oxygenase, hydroxylates the C-5 position of the indole ring, yielding protoviolaceinic acid, which is then transformed into violacein by VioC. It can convert proto deoxy violacein to deoxyviolacein when just VioC is present. In a spontaneous reaction, an imine dimer can produce chromopyrrolic acid, which is converted to Rebeccamycin, another kind of antibiotic by the enzymes RebP, RebC, RebG, RebM, and staurosporine, another antibiotic by the enzymes StaP, StaC, StaG, StaN, StaMA, StaMB.

In the first step of the violacein synthesis, the enzyme VioA (flavin-dependent tryptophan 2-monooxygenase), an orthologous enzyme of StaO and RebO, oxidizes L-tryptophan to generate indole-3 pyruvic acid imine (IPA imine) and reduces cofactor FAD to FADH. The IPA imine is then dimerized utilizing the enzymes heme-containing oxidase VioB, StaD, and RebD to form an unstable molecule imine dimer. Then, in a series of closely related initial steps, imine dimer can lead to the production of a variety of final compounds. By behaving as a catalytic chaperone employing a fold-related lipoprotein transporter, the enzyme VioE converts this unstable molecule into protodeoxyviolaceinic acid (PDVA) without the need for any cofactors or metals. VioD, a flavin-dependent oxygenase, hydroxylates the C-5 position of the indole ring, yielding protoviolaceinic acid, which is then transformed into violacein by VioC, which hydroxylates the C-2 position of the second indole ring and then undergoes oxidative decarboxylation (Hall and Mah, 2017). It can convert proto deoxy violacein to deoxyviolacein when just VioC is present (Antony et al., 2013). In a spontaneous reaction, an imine dimer can produce chromopyrrolic acid, which is converted to rebeccamycin, another kind of antibiotic by the enzymes RebP, RebC, RebG, RebM, and staurosporine, another antibiotic by the enzymes StaP, StaC, StaG, StaN, StaMA, StaMB (Devescovi et al., 2017). Violacein biosynthesis is well represented in a flow diagram (Figure 4).

Violacein activated the essential pathways related with immune and inflammatory response in toll like receptor (TLR) transfected HEK cell lines. hTLR8 receptor mediated signaling pathway is activated and not the hTLR7. *In silico* analysis depicted interaction of violacein with hTLR8 whose interaction is similar to imidazoquinoline compounds. CU-CPTga, an antagonist of hTLR8 shown to counteract the immunostimulatory effects of violacein (Venegas et al., 2019).

### Outer membrane vesicles

Outer membrane vesicles are the nano sized spherical component of the bacterial outer membrane that can range in size from 20 to 200 nm, dependant on the strain and environmental conditions (Reimer et al., 2021). OMVs are released from the bacterial outer membrane; thus, they actually contain proteins native to the outer membrane and periplasmic chemicals which are prevalent in the space between the two membranes (Rollauer et al., 2015). They are designated as OMV cargo since they serve the goal of a bacterial strain’s transport system. They are multipurpose cargo that benefits bacteria in a multitude of ways other than just transport. OMVs effectively remove hazardous toxins from bacterial cells when they are exposed to environmental or artificial stress (Jan, 2017). *C. violaceum* uses its OMV to thwart its rivals and kill them in an ecological niche using OMV-derived violacein, one of its potent antibiotics. OMVs can easily be incorporated into other organisms due to its rapid permeabilization into lipid bilayers (Wettstadt, 2020). The outer membrane of Gram-negative bacteria competitors, on the other hand, opposes the entry of violacein and prevents it from reaching the inner membrane. Because violacein is a hydrophobic molecule, *C. violaceum* transports it to competing bacteria through an aqueous media by using OMVs (Choi et al., 2020). *C. violaceum* CviI/CviR quorum system tailor the OMV secretion rate and OMV quantity depending on the cell population. Two vesiculation pathways, violacein biosynthesis and the VacJ/Yrb system act in opposite directions to modulate OMV secretion. Both vesiculation channels are QS-dependent, meaning they are triggered whenever the population density is high. However, the effect of vesiculation is totally reversed. The deletion of the *vioABCDE* operon resulted in a twofold reduction in vesiculation, demonstrating that violacein stimulates OMV biogenesis for delivery reasons. *VacJ* and *yrbE* deletion, on the other hand, resulted in an overabundance of vesicles. By regulating the *vioABCDE* operon and *yrbFEDCB*/*vacJ*, *cviI* and *cviR* regulate OMV synthesis. Other elements that control these vesiculation pathways include the bacterial envelope’s stress response protein and the peptidoglycan layer outer membrane binding responsive protein (Batista et al., 2020). Antibiotic resistance can be established by OMVs far more adequately than resistance mechanisms established at the genetic level, because OMVs can operate as a decoy by adhering to antibiotics and preventing it from reaching bacterial populations. Because OMV is an important driver of pathogenesis in *C. violaceum* infection, curbing its production is absolutely essential (Schwechheimer and Kuehn, 2015).

### Other virulence factors

In addition, the QS system regulates a multitude of virulence factors, comprising chitinase which breaks down chitin for the carbon source (Devlin and Behnsen, 2023) and target immune system components such as mucins and surface glycans, collagenase, cytolytic toxins (hemolysin and leukotoxins) which are detrimental to host cellular functions, exopolysaccharides of bacterial biofilms, flagellar proteins, lipases, metalloproteases, swarming motility, exoprotease synthesis, T2SS and T3SS (Miller et al., 1988; de Oca-Mejía et al., 2015). *C. violaceum* does indeed have type IV pili machinery, which is crucial for twitching mobility, bacterial aggregation and also host adhesion in addition to a single flagellum and as an outcome, escalating pathogenicity. The type IV pili machinery include known genes such as *pil B*, *C*, *D* and some other unknown genes (Galán et al., 2014). The functions of these genes were identified based on the comparative study with the *P. aeruginosa* type IV pili genes as they were nearly similar in assembly and characteristics. *C. violaceum*’*s* type VI secretion system (T6SS) which is regulated by the quorum-sensing mechanisms, is crucial for inter-competition amongst bacterial population. In the T6SS, there are roughly 14 core components. VgrG is one of the proteins that create holes in the host cells or competitive bacteria. Six *vgrG* genes are dispersed between *vgrG* islands and T6SS clusters in *C. violaceum*. The T6SS system is necessary for inter-bacterial competition but not for host infection. CviR, but not CviI, is an important QS protein that regulates T6SS. *VgrG3* is the most important of the six *vgrG* genes for regulating inter-bacterial competition (Previato-Mello et al., 2017). Other findings confirm that OhrR, a sensor of organic hydroperoxides, which is a component of the MarR, a winged-helix turn helix transcriptional family, is likewise significant for the pathogenicity of mice (Miki et al., 2011). In virulent strains, there will be an abundant amount of superoxide dismutase and catalase enzymes, which protect them from phagocytic attack and render them exceptionally virulent compared to avirulent strains (Du et al., 2016).

Other proteins involved in *C. violaceum* pathogenicity include hemolysin which helps in the lysis of blood cells in the systemic infections; outer membrane protein; collagenase which can cleave multiple sites at triple helical structure of collagen and destroys denatured collagen; flagellar protein which helps in the motility and pathogenicity; metallopeptidases which cleaves peptide bonds and helps in the degradation (Miller et al., 1988).

### T3SS system in *Chromobacterium violaceum*

**Figure 5:**
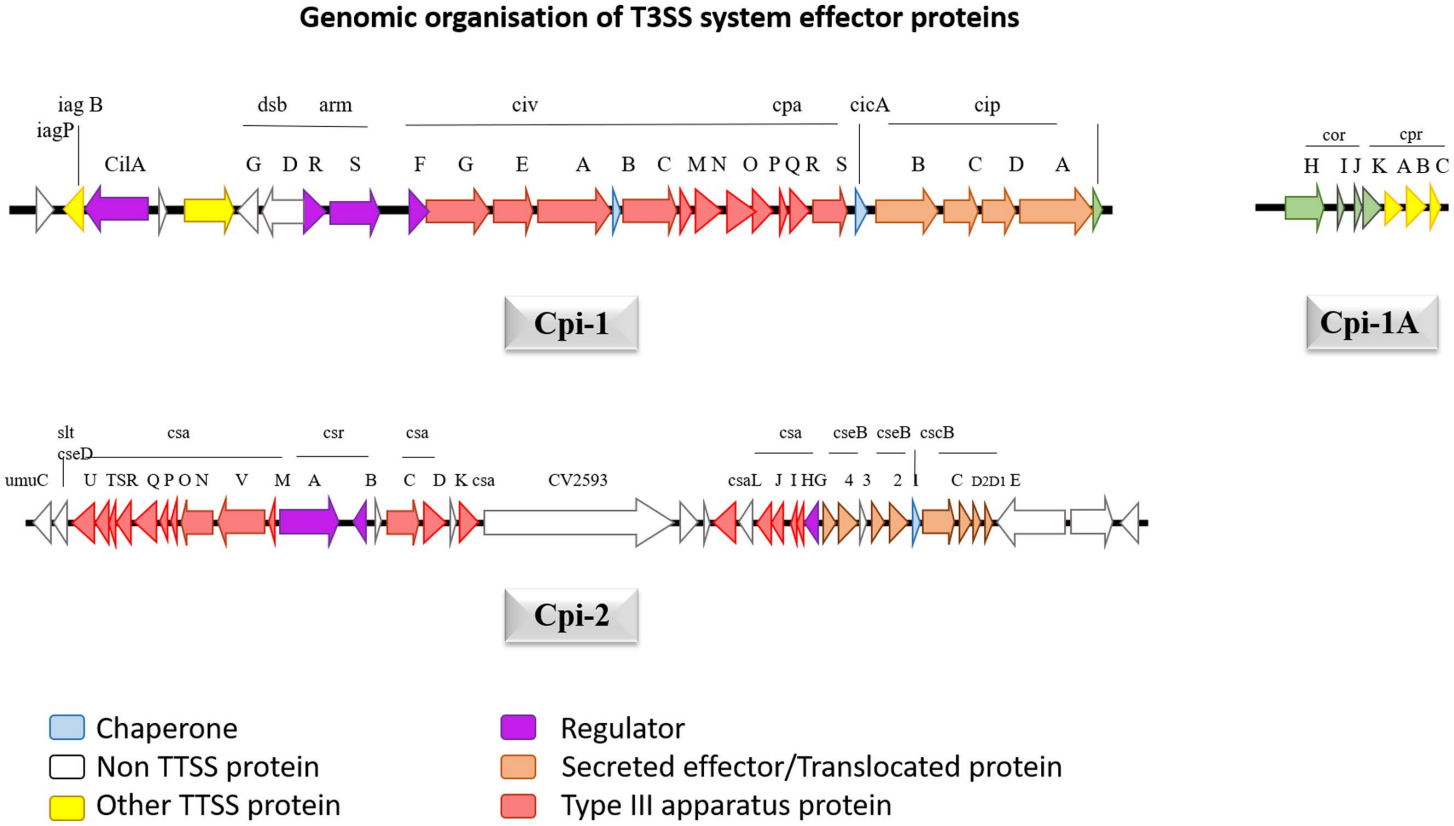
The genetic structure of Cpi-1, Cpi-1a and Cpi-2 pathogenicity islands of *C. violaceum* chromosome. Numbers indicate nucleotide positions based on *C. violaceum* sequencing data (Genome accession number: 1117).

Genomic sequencing for strain *C. violaceum* ATCC12472 was used to ascertain the pathogenicity. The results emphasize the existence of a numerous pathogenic components causative for *C. violaceum* infections in humans. The type 3 secretion system (T3SS), a multiprotein needle-like system that is exceptionally crucial in introducing the bacteria’s effector proteins into the host, which stimulates damages resulting from infection (Liu et al., 2022). The type 3 effector protein’s genomic organization was well described in Figure 5. Further investigation into the type 3 secretion system divulged that there are two primary types of T3SS namely *C. violaceum* pathogenicity island Cpi-1 and *C. violaceum* pathogenicity island Cpi-2. Cpi-1 and Cpi-2, are homologous to *Salmonella* pathogenicity islands Spi-1 and Spi-2 which encompass genes involved in harmonizing two kinds of T3SS proteins (Galán et al., 2014). Typically, these two islands would indeed be found on an adjacent location in *C. violaceum* genome. *Cpi-1* genes lie as two distinct clusters with one cluster coding for needle complex and the other gene makes up the cluster. *Cpi-2* genes are all grouped together in a single region. The primary reason for virulence is *cpi-1* and *cpi-1a*, as brought to light by deletion studies of the *cpi-1*, *cpi-1a*, and *cpi-2* secretion systems. The T3SS effector proteins Cpi-1 and Cpi-1a mediate the translocation of genes encoding other T3SS effectors. Although the functions of most T3SS-specific proteins are unknown, minimal research has been carried out to define the functions of *Chromobacterium* outer protein E (CopE), CivB, a putative chaperone specific for *copE* which were regulated by *cilA* [14]. Five putative regulators such as CilA, CivF, ArmR, SrB, SrC located within pathogenicity islands Cpi-1 and Cpi-2, whose mutagenesis and expression analyses further explained that CilA is master transcriptional activator for the significant number of the genes found in *cpi-1* and *cpi-1a* highlighting that it is a key regulator of T3SS genes (Batista and da Silva Neto, 2017).

The *cpi-1* and *cpi-1a* encode T3SS and are indispensable for hepatocyte cytotoxicity and cell death. *Cpi-1*/*1a* encodes 16 effector proteins that get translocated into hepatocytes, but their role is not clearly understood, leaving researchers with emerging research prospects (Alves de Brito et al., 2004). CopE, one of the effector proteins, functions as a guanine exchange factor (GEF) in Hela cells, activating NADH dehydrogenase subunit 3 homolog Rc1 and cell division control protein 42 homolog (Cdc42), allowing for actin configuration rearrangement and subsequent nonphagocytic epithelial cell invasion, which are responsible for *C. violaceum* pathogenicity in mice (Alves de Brito et al., 2004). *Cpi-2*’*s* precise and intricate function is uncertain, probably it may be involved in *C. violaceum*’*s* persistence after being engulfed by macrophages like that of *Salmonella*, given that most of the activities of T3SS in these two organisms are identical. Once phagocytosed *C. violaceum* successfully breakout from the phagosome to permeate the cytosol in epithelial cells, and the mechanism involves Cipc, a Cpi-1 translocating protein (Alves de Brito et al., 2004). The multiprotein complex of T3SS traverses the outer and inner bacterial membranes and use ATP as an energy source to release effector proteins to the cell’s exterior. Through a translocation system, T3SS has the ultimate capacity to transport those proteins straight into the cytoplasm of the host eukaryotic cells. *C. violaceum* T3SS effector protein Cpi-1 is located downstream of Cpi-2, which constitutes 26 genes from putative ORF 2615 to 2642. In a tRNA-leu gene area, the island terminates precisely at 183 bp downstream of gene 2642. Cpi-1 encodes for 31004 base pairs, with a G + C percentage of 67, which is extremely close to *C. violaceum*’*s* total GC content of 64.8 percent (Reimer et al., 2021). *Cpi-1a* is sandwiched between 2416 and gst 2424, which is 200 kb upstream of *cpi1* and encrypts 4190 bases with a GC content of 66 percent. Upstream of *cpi-1*, there are 39 putative open reading frames ranging from 2,574 to 2,614, which have been well delineated by the GC content reduction (54 percent) and is known as *cpi-2*. It contains 40,291 bp of genetic information. The *cpi-1* gene codes for the Inv-Spa transcriptional regulator and basal components, as well as the sis-sip spi-1 translocator operons.

### Immunological response to *Chromobacterium violaceum*’s type 3 secretion system

Cpi-1a corresponds to the spi-1 of prg—org operon, which encodes T3SS needle-like components which acts as a ligand to activate human immune responses. CilA is found to be the master regulator of *cpi1* and *cpi1a* expression, identified by transcriptional profiling of CipB DNA microarray. Cpi-1 and Cpi-1a regulatory molecule, is engaged in translocator-mediated pore construction in the host cell membrane, which is a vital element in *C. violaceum* cytotoxicity (Miki et al., 2010; Zhao et al., 2011). T3SS system can induce similar pathways in a few additional organisms, notably Pseudomonas, Salmonella, shigella and Legionella (Coburn et al., 2007). In humans and mice, the NAIP (NLR family apoptosis inhibitory protein) receptor recognizes *C. violaceum* T3SS Cpi-1 needle and rod proteins, and activate the NLRC4 (Nod-like receptor) inflammasome. Inflammasomes are cytoplasmic complexes that recognize bacterial infection and contribute to eradicating pathogens. Unlike other inflammasomes, such as NLRP1 and NLRP3, which have a wide range of activators, NLRC4 inflammasomes are operated by a limited number of activator molecules, most of which originate from bacteria, such as flagellin, T3SS, and a few T4SS (Yang et al., 2013). Human NAIP protein recognizes the Cpr1 needle subunit of *C. violaceum* T3SS, identical to mouse NAIP2 and NAIP5, which activates NRLC4 inflammasome in human macrophages, effectively combating *C. violaceum* infection (Vladimer et al., 2013; Zheng et al., 2020). In NLRC4, the signaling caspase activating and recruitment domain (CARD) is found in the N-terminal region, the NACHT/NOD domain is placed in the center part, and the leucine rich repeat (LRR) is located in the C-terminal region; therefore NLRC4 inflammasomes share a similar physical property with NLRP1 and NLRP3 inflammasomes. NACHT refer to a group of subdomains of the NLRC4 inflammasome which include the nucleotide-binding domain (NBD) and distinct helical domains. The interplay of the NACHT domain’s NBD and WHD keeps the NLRC4 inflammasome in a closed state (Duncan and Canna, 2018). The assembly of the NLRC4 inflammasome is similar to that of the apoptosome, which is an oligomer produced by apoptotic peptidase activating factor (APAF)-1. When NAIP binds to its ligand, three BIR domains are exposed, and its interaction with NLRC4 relieve LRR’s auto-inhibition resulting in pyroptosis (cell scorching), a type of programmed cell death (Vance, 2015).

**Figure 6:**
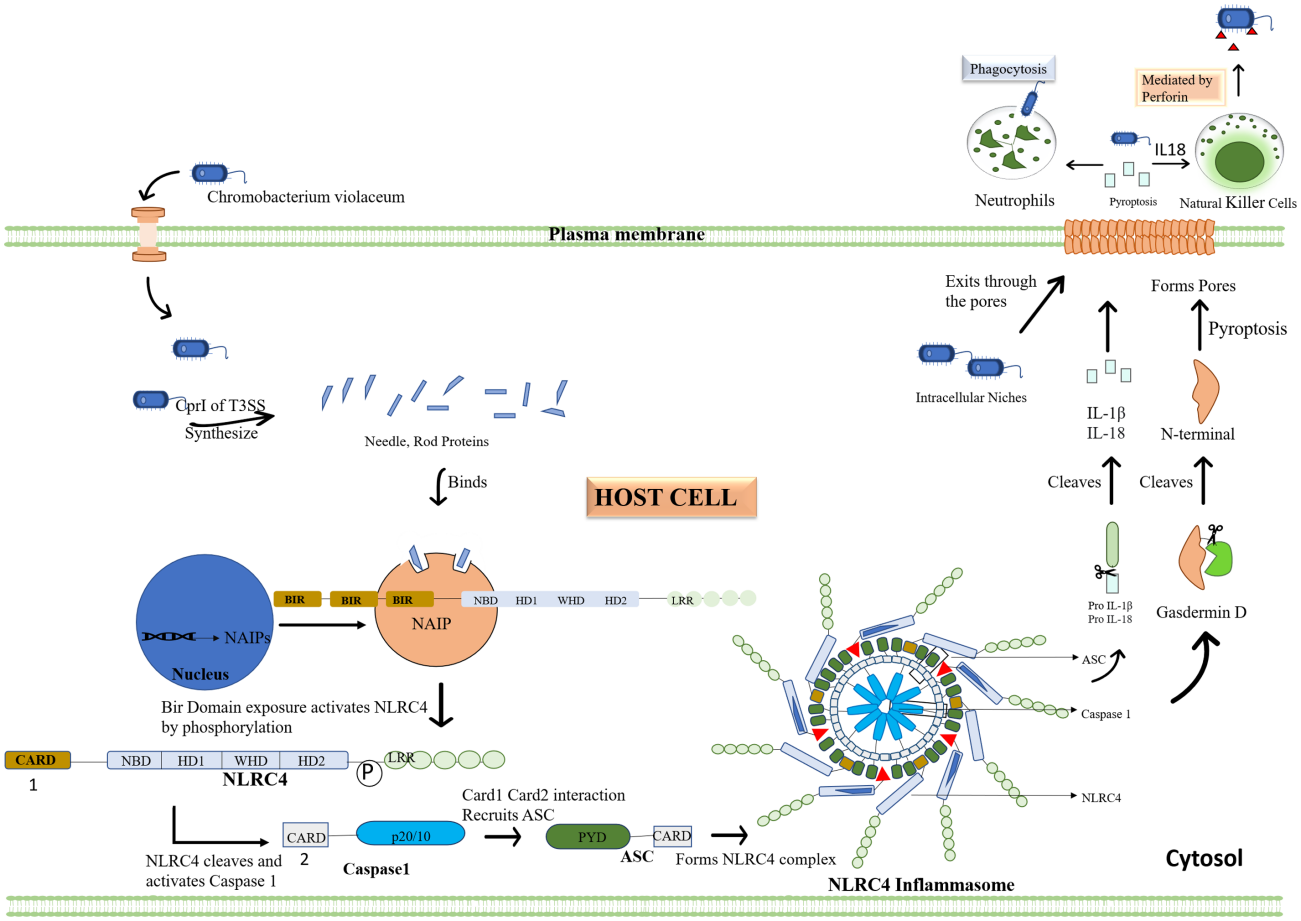
*Chromobacterium violaceum* or effector proteins infiltrate the cytoplasm of the host. The initial step of the inflammasome activation is when the type 3 secretion system’s CprI releases needle and rod protein, binding to the human NAIP. Then, activated NLRC4 cleaves and triggers caspase 1 in response to the exposed BIR domain. When NLRC4 and Caspase 1 engage, the Card domain attracts the ASC adapter, which together forms the inflammasome complex. Pro IL 1 and IL 18 are then converted to their active forms by the complex. By cleaving Gasdermin D, the inflammasome also activates it, establishing the plasma membrane pores via which interleukin and bacteria leave the cells. Other immune cells, such as neutrophils and natural killer cells, are drawn to the area by the interleukin, which acts as a signal. These cells eliminate the bacterial load in addition to the infected cell.

The full-length NLRC4 inflammasome is inert, but NLRC4 lacking the c terminal LRR is active, allowing caspase 1 and the pyroptosis pathway to be activated for efficient bacterial infection eradication (Sundaram and Kanneganti, 2021). Contrary to caspase 1, the caspase 11 pathway is not essential for *C. violaceum* clearance (Maltez et al., 2015). When the bacterial protein effector attaches to the human or mouse NAIP receptor protein, the NLRC4 inflammasome is activated along with another quiescent NLRC4 protein forming a self-propagating oligomer with a disc-like structure. As a result of caspase 1 activation, CARD-CARD interactions recruit adaptor protein ASC to NLRC4 and then bind caspase 1 to ASC, triggering pyroptosis. Caspase 1 cleaves and activates the pore-forming Gasdermin D which causes the inflammatory cell death of the infected host cell through pyroptosis by compromising membrane integrity. The PYD domain is absent in NLRC4 unlike NLRP3 and thus unaffected by K^+^ efflux. However, the CARD domain can interact with procaspase 1, causing pyroptosis and making the ASC molecule redundant in the NLRC4 inflammasome pathway. When the zymogen procaspase one is cleaved, it becomes activated into protease caspase 1, and it cleaves pro-IL-1β and pro-IL-18 into its active forms, which are then liberated outside the cell through a pore formed by Gasdermin D. Pyroptosis on its own is adequate for bacterial clearance in the spleen, whereas both pyroptosis and IL 18-driven NK cell-mediated clearance are required in the liver. In the liver, IL-1β is not as protective as IL-18. Perforin-mediated cytotoxicity is mediated by NK cells in the liver, and interferon is not required. Because of the diverse types of cells in the spleen and liver, different mechanisms are understandable. The inflammasome response mediated by innate immunity is sufficient for defense against *C. violaceum*; hence an adaptive immune response is not required (Maltez et al., 2015; Sundaram and Kanneganti, 2021). The immunological response in depicted with flow diagrams in Figure 6.

### Can palmitate act as an anti-quorum agent and immuno modulator simultaneously?

Some of the currently used medications against *C. violaceum* includes common antibiotics such as gentamycin, erythromycin and ciprofloxacin but the problem is the increasing antibiotic resistance over repeated irregular usage of antibiotics. Natural commodities are a renewable element that can be used for a multitude of purposes, including drug development. Natural products or derivatives of natural products account for 80 percent of all accessible medications (Veeresham, 2012). Half of the pharmaceuticals licensed for various ailments in the previous 20 years have a natural product backbone (Dias et al., 2012). The natural environment encompasses a broad range of bioactive metabolites that can act as antioxidants. Traditional medicine, which is primarily based on plant products, is now used by three-quarters of the world’s population. They can function as antimicrobial drugs, quorum sensing inhibitors, anti-inflammatory agents, and immunomodulators. Various strategies to intervene quorum sensing systems are collectively termed quorum quenching by which bacteria are made unable to communicate with one another. Quorum quenching can affect various processes that bacteria employ to establish infection, such as biofilm formation, toxin production, spore formation, and outer membrane vesicles (OMVs) release (Vadakkan et al., 2018).

Only 10 percent of total plant species have been investigated to date, therefore conducting research in that segment will improve the likelihood of enhancing the living standards of humans (Borges et al., 2016; Wylie and Merrell, 2022). The leaf extracts of *Ocimum sanctum*, the fruit extracts of *Passiflora edulis*, and the pseudo-stem extracts of *Musa paradisiaca* are all proved to be a versatile quorum-sensing inhibitors against *C. violaceum*. *P. edulis* bioactive molecule hexadecanoic acid, 2-hydroxy-1-(hydroxymethyl) ethyl ester interfered with quorum sensing system CviI and CviR of the *C. violaceum* and negatively regulated the quorum system (Musthafa et al., 2010; Venkatramanan et al., 2020). In *C. violaceum*, pigment and biofilm synthesis were reduced by hydroalcoholic extracts of *Tribulus terrestris* roots, with the highest effect at 2.5 mg/mL. The main compound was ß-1, 5-O-dibenzoyl ribofuranose, which interfered with the signaling activity of AHL molecules that regulate quorum sensing, rather than AHL production (Vadakkan et al., 2019). The root extract of *Desmodium gangeticum* showed anti-quorum sensing activity against *C. violaceum* by inhibiting the production of violacein when exposed at 300 μg/mL, thrice a day without any effect on bacterial viability. The main quorum quenching compound was identified as cis-3,5-dimethylocta-1,6-diene with significant bacterial silencing activity (Vadakkan et al., 2022). In a study by Vargas et al. (2021), found palmitic acid and phytol with efficient binding affinity for cviR receptor *insilico*, whereas *in vitro* study revealed phytol as an effective molecule inhibiting quorum sensing. *C. violaceum* infection in different animal model were efficiently treated with different antiquorum strategies. The survival of planarian flatform was enhanced by lactonase-mediated quorum quenching (QQ) against *C. violaceum* infection. Lactonase degraded the acyl-homoserine lactone (AHL) molecules that mediate quorum sensing (QS) in *C. violaceum*, thereby disrupting the bacterial communication and virulence. Planaria *Schmidtea mediterranea* succumbed to *C. violaceum* infection at a high dose of 4 × 10^9^ CFU/mL. However, QQ by lactonase significantly reduced the bacterial toxicity and increased planarian survival to 100 percent at the same load of *C. violaceum*, as reported by Mion et al. (2021). Kanekar and Devasya (2022) reported an increased survival rate of *C. violaceum* infected nematodes when they were subjected to the anti-quorum sensing molecule linalool. The nematodes, *C. elegans*, were pre infected with the pathogen *C. violaceum* and then administered with various concentrations of linalool, from 40 to 80 μg/mL. The findings indicate that linalool disrupted the quorum sensing mechanism of *C. violaceum* and attenuated its virulence factor secretion, thereby increased the viability of *C. elegans*. The survival of mice infected with *C. violaceum* was significantly enhanced by oral administration of essential seed oils from sunflower, chia and amaranth, according to a study by Macrina et al. through anti-quorum sensing activity by interfering with the bacterial communication and virulence. The mice treated with sunflower essential oil (EO) had a median survival of 18 h, followed by 16 h for chia EO and 14 h for amaranth EO. In contrast, the PBS control group had a median survival of only 10 h (Pérez-López et al., 2018). These studies provide evidence for the potential of anti-quorum sensing compounds to overcome of *C. violaceum* infection in various animal models.

Immune system functions and efficiency are influenced by a variety of exogenous and endogenous substances known as immunomodulators. Alkaloids, diterpenoids, flavonoids, glycosides, lactones, and polysaccharides derived from plants can act as immunostimulants, immunoadjuvants, and immunosuppressants by enhancing the efficacy of immune system components or mediators, improves the efficacy of vaccines or drugs, and downregulate the immune system, respectively (Di Sotto et al., 2020). Cytotoxic synthetic medications can also be used for this purpose, but they are accompanied by a number of adverse side effects for the host, as well as being prohibitively expensive for commercial reasons. Palmitic acid is one of the natural bioactive which is majorly found in plant species. Using palmitate, Lie et al. described non-pathogenic NLRC4 inflammasome activation. Palmitate promoted apoptosis in astrocytes via the NLRC4 inflammasome. Caspase is activated, and an inflammasome complex containing CARD and ASC are recruited, resulting in the development of IL 1β in astrocytes (Wen et al., 2021). Palmitate treatment in Hep G2 human hepatomal cells significantly increased the production of pro inflammatory cytokines such as IL-1β, IL-18, TNF-α and MCP-1. The observed active form of above mentioned cytokine was assumed to be because of activated NLRC4 inflammasome which was further proved by elicited mRNA expression of NLRC4 protein. The above-stated higher expression was dose-dependent in nature, i.e., in the lower dose of palmitate the NLRC4 inflammasome is expressed less than in the higher dose. To elicit increased NLRC4 mediated defense against *C. violaceum* palmitate can be used in a lower dose (Luo et al., 2012). Palmitic acid at the concentration of 1 mM is claimed to suppress violacein synthesis in *C. violaceum* by around 50% while only disrupting growth by about 5% (Pérez-López et al., 2018). *In vivo* studies monitoring this palmitic acid-containing essential oils delayed the death of *C. violaceum* infected mice. This stimulates the research notion that palmitic acid can be employed in dual mode treatment for *C. violaceum* infections, possibly through its antiquorum and NLRC4 upregulation abilities, which will be efficient in clearing the infected cells. Here the proposed idea is to use palmitate as dual therapeutic strategy against the *C. violaceum* and similar kind of infections. The concentration to attain both the effects should be standardised using experimental procedures.

## Future perspectives and conclusion

**Figure 7:**
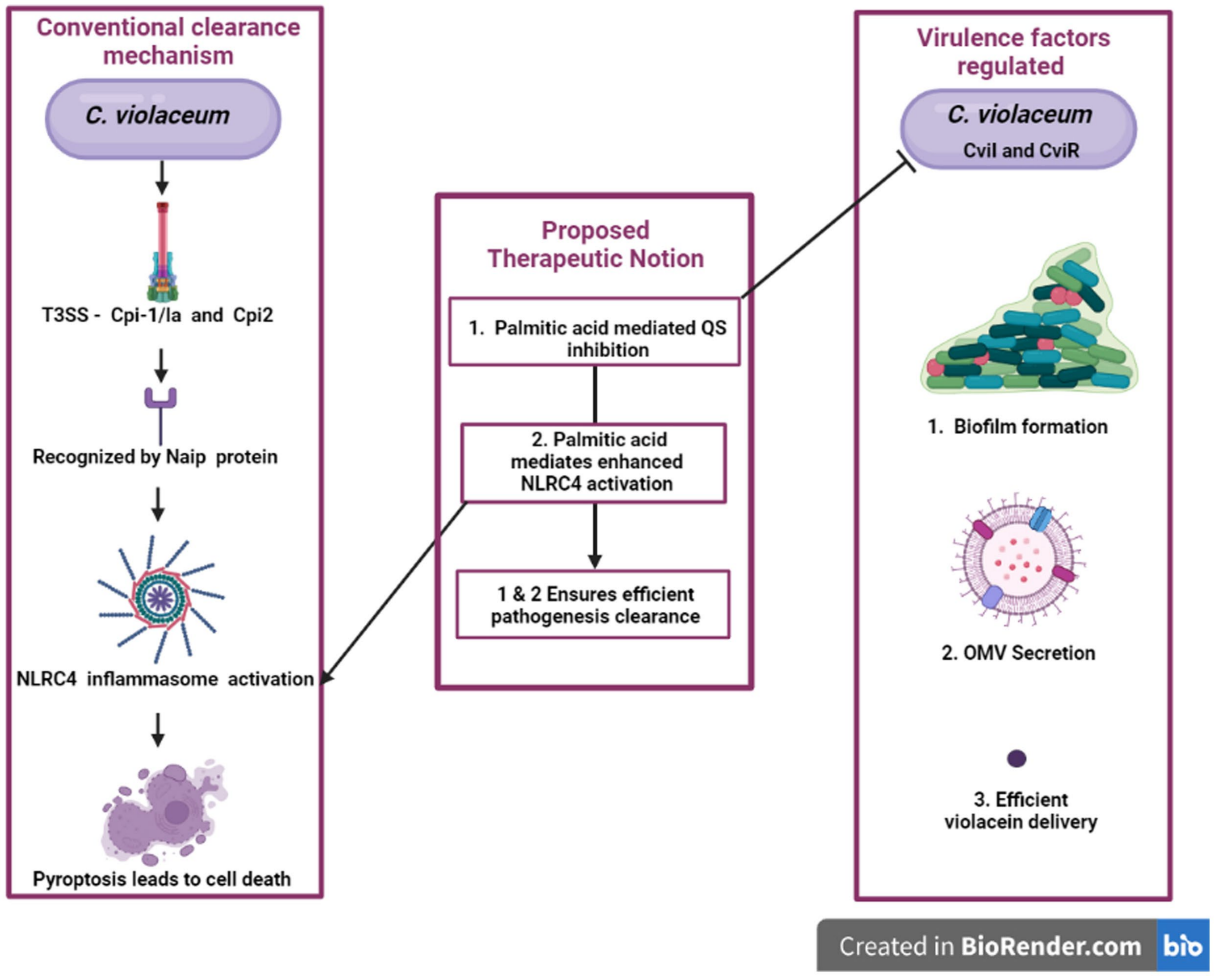
An innovative approach’s graphical representation “A possible way to reduce the harmful effects of *C. violaceum* in systemic infections is to target two aspects of its pathogenesis: the quorum sensing system that regulates its virulence factors and the NLRC4 inflammasome that modulates the host’s immune response. By disrupting the quorum sensing signals that coordinate the production of toxins, pigments, and biofilms in *C. violaceum*, we can impair its ability to cause damage and evade defenses. By activating the NLRC4 inflammasome in the host cells, we can enhance the clearance of intracellular *C. violaceum* by inducing pyroptosis, a form of inflammatory cell death. This dual strategy may offer a novel and effective way to combat *C. violaceum* infections.”

It is reported that, when NLRC4 inflammasome is overexpressed in macrophages for a small load of *S. typhimurium*, it is very efficient in clearing the pathogen, but when the bacterial load increases, overexpression of NLRC4 inflammasome causes cell toxicity which is detrimental to the host (Wen et al., 2021). For any microbial infection, we can represent a particular or a combination of natural compounds to restrict the microorganisms’ quorum sensing, rendering them less virulent thereby enabling our immune system to efficaciously eradicate before any harm. Furthermore, if that combination of natural products is effective in immunomodulation, i.e., boosting inflammasome-mediated pyroptosis, the natural product’s efficacy will explode. If we are just reduce pathogenicity by blocking signals in the anti-quorum sensing treatment, bacteria still exist in the host with the possibility of gaining virulence due to host susceptibility for other infections or mutation. Natural product-based therapeutics will be immensely beneficial if we can effectively prevent virulence development by blocking QS and also by promoting NLRC4 inflammasome-mediated cell lysis of infected cells. Palmitate is a compound reported to have both anti-quorum activity and NLRC4 inflammasome activating properties (Liu and Chan, 2014; Pérez-López et al., 2018). Hence compounds like it can be effectively utilized to do both jobs simultaneously for better treatment which will be a novel treatment strategy to fight against antimicrobial resistance development. The idea is represented by a flow diagram in Figure 7. The advantage behind this anti-quorum mediated proposed therapeutic technique is that this can be an alternative to antimicrobial therapy which may bypass the resistance development. Anti-quorum sensing reduces the expression of virulence factors without affecting the bacterial survival, thus avoids strong Darwinian selective pressure exhibited by antibiotics. These therapeutics can render any pathogenic bacteria less virulent hence reduce the severity of the infections. The take home point we are trying to convey here is we need to identify a single phytochemical compound with multiple treatment efficiencies against any infectious pathogen. Here the proposed palmitate is one such phytochemical with this potential, because palmitate can trigger NLRC4 inflammasome pathway which will heighten the pathogen clearance mechanism along with antiquorum propensity against *C. violaceum*. The limitation to this proposed idea exists in identifying a specific phytochemical against individual pathogenic organisms.

## Author contributions

MV: Writing – original draft. EN: Writing – review & editing.
